# Mitochondrial abnormalities: a hub in metabolic syndrome-related cardiac dysfunction caused by oxidative stress

**DOI:** 10.1007/s10741-021-10109-6

**Published:** 2021-05-05

**Authors:** Aoxue Li, Ningning Zheng, Xudong Ding

**Affiliations:** 1 Department of Pharmaceutical Biotechnology, China Medical University-The Queen’s University of Belfast Joint College, China Medical University, Liaoning, China; 2 Covance Pharmaceutical Research and Development (Beijing) Co, Ltd, Beijing, China; 3 Department of Pathophysiology, College of Basic Medical Science, China Medical University, Liaoning, China; 4 Department of Anesthesiology, Shengjing Hospital, China Medical University, Liaoning, China

**Keywords:** Oxidative stress, Mitochondria, Reactive oxygen species, Metabolic syndrome, Cardiac dysfunction

## Abstract

Metabolic syndrome (MetS) refers to a group of cardiovascular risk elements comprising insulin resistance, obesity, dyslipidemia, increased glucose intolerance, and increased blood pressure. Individually, all the MetS components can lead to cardiac dysfunction, while their combination generates additional risks of morbidity and mortality. Growing evidence suggests that oxidative stress, a dominant event in cellular damage and impairment, plays an indispensable role in cardiac dysfunction in MetS. Oxidative stress can not only disrupt mitochondrial activity through inducing oxidative damage to mitochondrial DNA, RNA, lipids, and proteins but can also impair cardiomyocyte contractile function via mitochondria-related oxidative modifications of proteins central to excitation–contraction coupling. Furthermore, excessive reactive oxygen species (ROS) generation can lead to the activation of several mitochondria apoptotic signaling pathways, release of cytochrome c, and eventual induction of myocardial apoptosis. This review will focus on such processes of mitochondrial abnormalities in oxidative stress induced cardiac dysfunction in MetS.

## Introduction

**Fig. 1:**
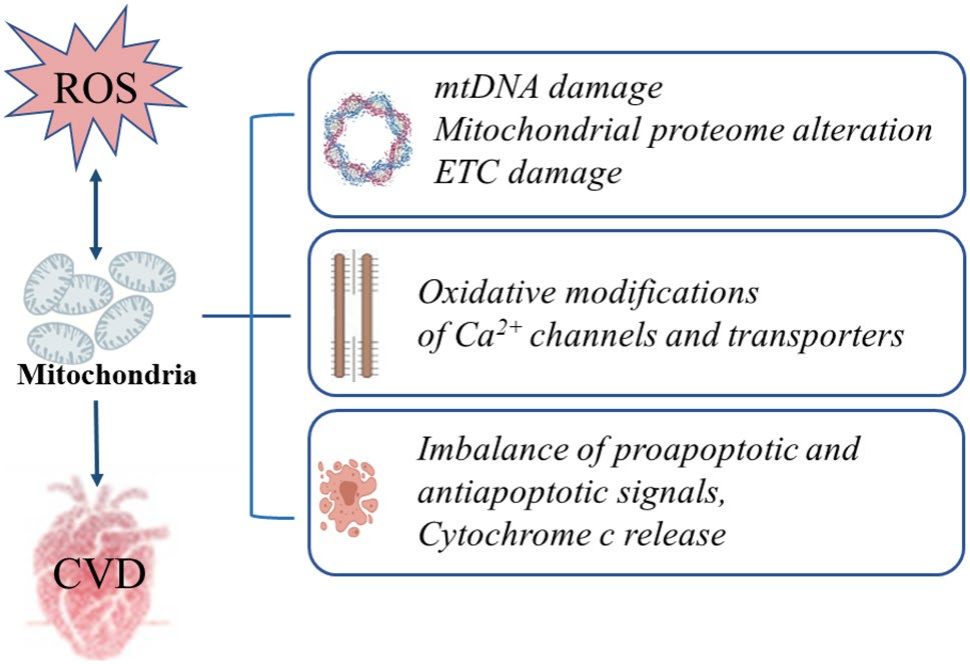
Mitochondria act as a key regulator in metabolic heart disease caused by oxidative stress. Three possible mechanisms of mitochondria involving in this process are summarized: ROS mediate oxidative damage to mitochondrial DNA, RNA, and proteins, resulting in ETC function impairment. The mitochondria-related oxidative modifications of ${Ca}^{2+}$ channels and transporters can directly cause cardiomyocyte systolic and diastolic dysfunction. Excessive ROS induce mitochondria-dependent cardiomyocyte apoptosis by activating proapoptotic signaling pathways, suppressing antiapoptotic signaling pathways, and mediating cytochrome c release. ROS reactive oxygen species, CVD cardiovascular disease, mtDNA mitochondrial DNA, ETC electron transport chain

Metabolic syndrome (MetS) refers to a group of cardiovascular risk factors comprising insulin resistance, obesity, dyslipidemia, increased glucose intolerance, and increased blood pressure [1]. MetS components can act independently as risk factors for cardiovascular disease (CVD), while their combination may significantly increase the morbidity and severity of cardiovascular conditions. When cardiac dysfunction occurs in MetS patients with coronary artery disease or other etiologies, it suggests that they may suffer specific cardiomyopathies, including obesity-related and diabetic cardiomyopathy and insulin resistance-related cardiac dysfunction [2]. Controlling such cardiovascular-related injuries is an important strategy to alleviate MetS clinical mortality. A state of elevated oxidative stress appears to be a central mechanism underlying the pathophysiology of MetS and related CVD [2]. Excessive reactive oxygen species(ROS) induce irreversible structural and functional impairment in cardiac myocytes, involving multiple pathological cardiovascular conditions, and also been widely known as a key contributor for cardiac hypertrophy and remodeling through stimulating signal transduction which controls cardiomyocytes apoptosis and necrosis. The key role of mitochondria in the production of ROS and regulation of cardiomyocyte survival and heart pump function determines that mitochondria dysfunction is a great stimulus triggering almost all pathological aspects of oxidative stress induced heart dysfunction. Medium and later stage of cardiac hypertrophy with decreased amounts of mitochondria usually accompanies with contractile dysfunction [3]. Mitochondrial structure damage and function decline form a catastrophic cycle with ROS and continue to advance the progression of the disease. In this review, we are just on the perspective of this hub, mitochondria, to summarize recent insights into the mechanisms of oxidative stress-related cardiac dysfunction from mitochondrial damage, cardiomyocyte contractile dysfunction, and apoptosis (Fig. 1).

## Oxidative stress and mitochondrial damage

Heart is a high-energy demand organ with extremely dependence on the normal structure and function of mitochondria [4]. Mitochondria represent a primary source of ROS and also suffer ROS-mediated damage, suggesting that a pathophysiological relationship exists between oxidative stress and mitochondrial dysfunction [5, 6]. Maintaining ROS generation and removal homeostasis is a pivotal prerequisite for ensuring mitochondria metabolic adaption. In turn, the pathological process of excessive ROS is characterized by mitochondrial abnormality, including enhanced peroxidation of mitochondrial lipids, decreased mitochondrial DNA (mtDNA) and mitochondrial RNA (mtRNA) copy numbers, and the reduced oxidizing ability [7].

### Mitochondrial DNA damage

Oxidative stress has destroyed mitochondrial macromolecules both at and near the sites of their formation, specifically induced oxidative damage to mtDNA, mtRNA, lipids, and proteins, which eventually contributes to mitochondrial dysfunction [7]. Mitochondria have their own DNA which encodes 13 subunits of mammalian respiratory complexes, as well as 22 tRNAs and 2 rRNAs [8]. mtDNA exhibits a higher sensitivity to oxidative damage than nuclear DNA, possibly because mtDNA is closer to ROS-generating respiratory chains, while also lacking the protection of histone-like proteins and the ability to repair DNA damage [9]. Excessive ROS production in mitochondria can progressively destroy mtDNA, leading to a concomitant reduction in mitochondrial RNA and protein levels, and eventually to the loss of mitochondrial function [10]. This is also supported by in vitro studies on cultured vascular endothelial and smooth muscle cells, which demonstrated that excessive ROS generation can lead to mtDNA damage, gene expression alterations, and mitochondrial dysfunction [10]. Regarding mtDNA damage, ROS accumulation has a higher propensity to induce the generation of basic sites and strand breaks than mutagenic base lesions, which may be related to the ability of mitochondria to retain their genetic integrity. If mitochondrial base excision repair pathway components fail to correctly repair mtDNA damage by removing the damaged DNA bases and abasic sites, mtDNA will become unstable and degraded, eventually resulting in mitochondrial dysfunction [11]. Specifically, superoxide anions (O^2•*−*^), one of the major ROS, are produced by the mitochondrial electron transport chain (ETC) through the reduction of oxygen [12]. These anions are then released into the mitochondrial matrix and intermembrane space and converted either spontaneously or enzymatically to hydrogen peroxide (${H}_{2}{O}_{2}$). Finally, ${H}_{2}{O}_{2}$ diffuses throughout the cell and generates highly reactive hydroxyl radicals with a strong capacity to damage mtDNA [13]. Studies have shown that the main mtDNA modifications resulting from ROS-mediated insult are the generation of 7,8-dihydro-8-oxo-2-deoxyguanosine (8-oxoG) among purines [14] and thymine glycol among pyrimidines [15]. In addition, excessive mitochondrial ROS generation may result in a vicious cycle of declining mitochondrial function, whereby mitochondrial ROS causes mtDNA damage and mutations, which conversely exacerbate the injury to respiratory chain function and induce subsequent ROS accumulation. The decreased mtDNA copy number and subsequent mtDNA defects due to excessive ROS production lead to a decline in mitochondrial transcript and protein levels, as well as the impairment of ETC function [7]. Consequently, mitochondrial dysfunction contributes to increasing mitochondrial oxidative injury, impairing mitochondrial respiration, and disturbing mitochondrial substrate utilization, which may eventually lead to cardiac dysfunction.

### Mitochondrial proteome alteration

Alteration of the mitochondrial proteome and associated mitochondrial dysfunction is implicated in heart disease. It has been demonstrated that an underlying connection exists between oxidative stress and changes in the cardiac mitochondrial proteome [16], although the relationship has yet to be extensively investigated. In a rat streptozotocin model of chronic type I diabetes, myocardial oxidative stress and concomitant proteome variations in diabetic hearts were shown to proceed synchronously, and 50% of proteins exhibiting altered expression were localized to mitochondria [16]. Additionally, a different study conducted by Dai et al. also reported the central roles of mitochondrial oxidative stress in proteome remodeling under heart failure induced by pressure overload, with dramatically altered 96 proteins like reduced fatty acid metabolic proteins and elevated glycolytic enzymes in the cardiac ventricular tissues of wild type mice with transverse aortic constriction (TAC)-induced heart failure compared with wild-type controls. Among them, 28% changed proteins are metabolic proteins which are mainly involved in fatty acid metabolism, like Acyl-CoA dehydrogenases; 23% of changed proteins are ETC proteins; and 30% of the proteome remodeling is comprised by proteins participated in transmembrane transport, ATP generation, protein folding, and proteolysis, as well as apoptosis [17]. Interestingly, regarding the changes of ETC complexes, the abundance of various subunits was observed to change inconsistently [17]. The inconsistency was also previously reported by Bugger et al. that over 50% of ETC proteins decreased with some subunits of complexes I, II, and V increased after TAC-induced heart failure [18]. In contrast, Dai et al. found that the increased subunits are more than decreased ones after TAC, and suggested that the increased abundance of these ETC components seems to lead to more dysfunctional damaged proteins [17], which is in opposition to most studies considering the declined mitochondrial respiratory function in heart failure is associated with susceptibility of ETC complexes to oxidative damage. However, mitochondrial catalase-mediated scavenging of mitochondrial ROS was demonstrated that can greatly attenuate the mitochondrial proteome remodeling in response to TAC [17]. Therefore, an increased understanding of the relationship between mitochondrial proteomic changes and oxidative stress may be important for predicting and analyzing the therapeutic response to both established and novel antioxidant therapeutics for the treatment of heart failure. When cardiovascular disease occurs, the transformation of metabolic substrates during the process of mitochondrial energy metabolism, changes in metabolic mode, and related mitochondrial metabolomics modification changes involved in these processes will be further clarified with the in-depth analysis of sequencing technology.

## Oxidative stress and mitochondria-dependent heart pump dysfunction

The normal contraction and relaxation of cardiomyocytes depend on the appropriately adjusted calcium ions level in the cytoplasm, the combined state of calcium and troponin, and the formation of cross bridges. Excessive oxidative stress can directly cause cardiomyocyte systolic and diastolic dysfunction by inducing oxidative posttranslational modifications of proteins involved in excitation–contraction coupling (ECC) in MetS hearts [19]. ECC describes the conversion of electrostimulation, such as an action potential, to a mechanical response in a single cardiomyocyte. In this accurate tuned process of electrostimulation, intracellular sodium ions ($${Na}^{+})$$ and calcium ions ($${Ca}^{2+})$$ play central roles [20]. Deficient $${Ca}^{2+}$$ and $${Na}^{+}$$ handling has been suggested to result in oxidative stress by initiating a vicious cycle that affects ECC and leads to cardiac dysfunction and heart failure [21]. Mitochondrial calcium uptake plays a very important role in maintaining the normal physiological functions of cells, including stimulating ATP production, inhibiting autophagy, rectifying intracytoplasmic calcium signals, and regulating cell death. Mitochondrial calcium uniporter (MCU) are important mediators of mitochondrial calcium integration signals. Studies suggest that the regulation of mitochondrial calcium uptake 1 (MICU1) and mitochondrial calcium uptake 2 (MICU2) on MCU activity involves a gating mechanism: when the cell is in a resting state and the concentration of $${Ca}^{2+}$$ in the cytoplasm is low, MICU1-MICU2 inhibit $${Ca}^{2+}$$ from entering the mitochondria through the MCU. When the cell is stimulated by signals and the concentration of $${Ca}^{2+}$$ in the cytoplasm rises and exceeds a certain threshold (about greater than 1 μM), MICU1-MICU2 allow $${Ca}^{2+}$$ to enter the mitochondria through the MCU. This gating mechanism not only effectively prevents mitochondria from ingesting excessive $${Ca}^{2+}$$ in the resting state of the cell, leading to oxidative damage and even cell death, but also enables mitochondria to respond to and rectify cytoplasmic calcium signals to complete normal cellular physiological processes. Heart disease has been found to be related to mitochondrial calcium disorders. Ming-Feng Tsai’s study shows that the formation of MCU complex may contribute to its localization at the mitochondrial inner and outer membrane contact point, thereby promoting mitochondrial uptake of $${Ca}^{2+}$$ from the cytoplasm, which elucidates the molecular mechanism by which mitochondria regulate calcium uptake in response to cytoplasmic calcium signals [22]. Sarcoplasmic/endoplasmic reticulum calcium ATPase 2a (SERCA2a), which is expressed in cardiac muscle and controls its relaxation through sequestering $${Ca}^{2+}$$, displays impaired activity due to increased oxidative stress in the MetS heart, which eventually leads to cardiac diastolic dysfunction. The underlying mechanism was shown to be that posttranslational modification of SERCA2a induced by ROS overproduction results in prolonged $${Ca}^{2+}$$ transients and slower SERCA2a-mediated $${Ca}^{2+}$$ reuptake [23]. ROS accumulation gives rise to cardiac $${Ca}^{2+}$$ overload through redox modulation of ion channel and ion pump activity [24]. Regarding the targets for oxidative modifications, the sulfhydryl groups of cysteine residues have been shown to be critical targets for oxidative modification. The oxidative posttranslational modifications can elicit diverse functional results depending on the type of pump and ion channels, as well as other transporter types. For instance, the redox modification of thiol groups can hyperactivate cardiac ryanodine receptor (RyR) which is a redox-sensitive sarcoplasmic reticulum $${Ca}^{2+}$$ release channel located in the inner mitochondrial membrane (IMM) [25, 26], leading to calcium leak, but it can also inhibit SERCA activity [27], thereby directly changing calcium kinetics. As a result, the altered calcium kinetics disrupt the mechanisms of force activation, generation, and transmission. In addition, mitochondrial $${Ca}^{2+}$$ overload is associated with elevated ROS release and decreased ATP generation in heart failure. Specifically, intramitochondrial calcium contributes to maintaining energy metabolism homeostasis and cardiac function via activating Krebs cycle dehydrogenases; however, this process is disrupted in heart failure, and the impaired mitochondrial $${Ca}^{2+}$$ uptake results in NADPH oxidation, excessive ROS production, and ultimately cardiac dysfunction [21]. On the other hand, pirfenidone has been demonstrated that it cannot only enhance the contractility of cardiomyocytes by promoting myofilament $${Ca}^{2+}$$ desensitization and increasing $${Ca}^{2+}$$ uptake but also inhibiting ROS generation so as to attenuate oxidative stress [28]. Hence, oxidative stress plays an indispensable role in impairing cardiac contractility through oxidative modifications of ion channels, pumps, or other transporter types, which alter their activities and eventually lead to impaired ECC.

## Oxidative stress and mitochondria-dependent cardiomyocyte apoptosis

Oxidative stress is assumed to be one of the main risk factors for triggering cardiomyocyte death through apoptosis or necrosis and is involved in the pathogenesis of apoptosis through regulating downstream signaling pathways [29]. The changes of mitochondrial permeable transduction pore (mPTP) and membrane potential are an important early signal of oxidative stress response. The occurrence of oxidative stress and the consumption of ATP can induce mPTP long opening in the mitochondrial intima, and the decrease of proton gradient and potential energy, resulting in severe swelling of the mitochondria and the release of large amounts of cytochrome c and apoptosis-inducing factor, thus stimulating both caspase-dependent and caspase-independent cascade apoptosis reaction, eventually promoting the occurrence and development of cardiovascular diseases. Maintaining the integrity of mitochondria and preventing the expression of apoptotic genetic programs are a feasible treatment for cardiomyocyte apoptosis. Oxidative stress-induced mitochondria-dependent apoptosis in cardiomyocyte is thought to involve three mechanisms: the activation of proapoptotic signaling pathways; suppression of antiapoptotic signaling pathways; and exerting an immediate ROS-induced effect on mitochondria that leads to the release of cytochrome c.

### Mitochondria-related proapoptotic signaling

Oxidative stress has a role in the pathogenesis of apoptosis as it can rapidly activate proapoptotic signaling pathways. Fluctuations in ROS generation have been demonstrated to activate a set of apoptotic signaling regulatory proteins, thereby mediating cellular responses [30]. Numerous studies have demonstrated that mitogen-activated protein kinases (MAPKs), c-Jun N-terminal kinases (JNKs), and p38 MAPK, regulator signals for cellular proliferation, differentiation, and survival, can be activated by stress and play crucial roles in mitochondria signal transduction during oxidative stress-induced apoptosis. There is also sufficient evidence to consider the possibility of targeting these signals-mitochondrial interactions in the prevention and treatment of heart disease. In the JNK and p38 pathways, initially, signal-regulating kinase 1 (ASK1), which is activated by oxidative stress, acts as a mitogen-activated protein kinase kinase kinase (MAPKKK) to phosphorylate and directly activate respective mitogen-activated protein kinase kinases (MAPKKs) of JNK and P38; these subsequently phosphorylate and activate JNK and p38, which then directly or indirectly regulate downstream apoptosis-related targets [31]. The p38/JNK and Janus kinase 2 (JAK2)/signal transducer and activator of transcription 3 (STAT3) pathways can not only be activated by ROS but also increase ROS generation, which is related to the elevated phosphorylation levels of p38/JNK and JAK2 [32]. Inhibition of mitochondrial JNK signal can attenuate the effect of ROS and mitochondrial dysfunction, thereby reducing the scope of myocardial infarction after ischemia–reperfusion [33]. ROS and JNK-dependent activation of mitochondrial proapoptotic pathway dominate the cardiomyocyte apoptosis induced by β-adrenergic receptor stimulation [34]. Moreover, spermine, an antioxidant, can effectively inhibit excessive oxidative stress and reduce cardiomyocyte apoptosis under high-glucose conditions through significantly suppressing the activation of the p38/JNK and JAK2 pathways and results in decreased ROS generation, which can, conversely, suppress the phosphorylation of p38/JNK and JAK2 and eventually reduce ROS accumulation [32]. In addition, ASK1 in mitochondria can also act independently of JNK and P38 activation to induce apoptosis after being activated by oxidative stress [35]. Consequently, the JNK, p38, and ASK1 pathways all act as inducers of mitochondria-dependent apoptosis when activated by oxidative stress.

### Mitochondria related antiapoptotic signaling

The imbalance between proapoptosis proteins and antiapoptosis protein has been demonstrated as the dominant cause for myocardial apoptosis related to oxidative stress [36]. Regarding the mitochondrial antiapoptotic signaling pathways, firstly, PI3K/AKT pathways, which is crucial for glucose uptake and insulin sensitivity, has been reported with markedly deactivation in high-fructose diet-induced metabolic syndrome [37]. The PI3K/AKT pathway has also been demonstrated as a vital antiapoptotic signaling pathway for cardiomyocytes in heart failure to attenuate myocardial apoptosis. To be more specific, the PI3K-AKT pathway of cardiomyocytes is activated by hypoxia; then, pAKT is transferred from the cytoplasm to the mitochondria, resulting in a significant increase of cytochrome c oxidase activity, thereby inhibiting cell apoptosis [38]. In a study of the effect of high fructose on cardiac apoptosis and survival pathways, it was found that compared with the control group, rats in the fructose-induced metabolic syndrome group (FIMS) had more apoptotic cells, and activated cytoplasmic cytochrome c, caspase-3 and 9 levels (mitochondrial pathway) significantly increased; p-PI3K, p-Akt, and Bcl-2 protein levels significantly decreased. Activated cardiac mitochondrial-dependent apoptosis pathways have taken an important part of the responsibility in the pathogenesis of high-fructose diet-induced heart failure [39]. The activation of PI3K/AKT signaling pathway also results in phosphorylation of the downstream signaling like glycogen synthase kinase-3β (GSK3β) and Nrf2 [36]. Subsequently, the PI3K/AKT/GSK3β pathway has been pointed out the crucial roles in acute myocardial infarction to inhibit cardiomyocyte apoptosis by increasing expression of antiapoptosis protein, B‑cell lymphoma‑2 (Bcl-2), and reducing the expressions of proapoptosis proteins including Bax and cleaved-caspase 3 [40]. A recent study, which was conducted with Sprague Dawley rats suffering heart failure after acute myocardial infarction, indicated that Qiliqiangxin capsule, a traditional Chinese medicine with cardioprotective effects, seems to maintain cardiac function and protect cardiomyocytes from mitochondria-dependent apoptosis induced by oxidative stress through activating the PI3K/AKT signaling and upregulating phospho-GSK3β [36]. Secondly, the MAPK/extracellular signal-regulated kinases (ERK1 and ERK2) signaling pathway is also involved in cardiac protection by preventing myocardial cell apoptosis in myocardial ischemia/reperfusion injury via the activation of pathway [41]. For example, DJ-1, as a multifunctional protein with antioxidant properties, exerts cytoprotective effects under conditions of oxidative stress by mediating cell survival and proliferation through activating the ERK1/2 pathway, as well as by suppressing apoptosis through the inhibiting of ASK1 activation [42]. Deng and Kang’s studies showed that the activated ERK1/2 colocalize with mitochondria, and then phosphorylate Bcl-2 and Bad, thereby participating in the antiapoptotic effect mediated by mitochondria [43, 44]. Thus, it is likely that oxidative stress suppresses these mitochondria-regulated antiapoptotic signaling pathways and leads to the imbalance between proapoptotic signaling and antiapoptotic signaling, resulting in cardiomyocyte apoptosis. However, the existing researches exploring mitochondria antiapoptotic signaling principally focus on the model of myocardial infarction. The models such as MetS-related CVD and diabetic cardiomyopathy need to be adopted more widely to observe the alteration and roles of antiapoptotic signaling in further research.

### The release of cytochrome c

Cytochrome c, an evolutionarily conserved and nuclear-encoded mitochondrial protein, is essential not only for energy production but also for apoptosome formation and apoptotic cascade [45]. Regarding the specific mitochondrial-dependent apoptosis process, cytochrome c firstly separates from cardiolipin, the membrane-anchoring lipid to which cytochrome c is tethered, and then released into the extramitochondrial environment via mitochondrial permeability pores following the permeabilization of the outer mitochondrial membrane. Once released into the cytosol, cytochrome c complexes with dATP and apoptotic protease-activating factor 1 (APAF-1), resulting in an increase in the affinity of the complex for dATP, oligomerization of APAF-1, and subsequent apoptosome formation. As soon as the complex is formed, caspase-9 is activated, which initiates the hydrolytic cascade and leads to the activation of the effector caspases, and, eventually, to apoptosis [46]. Excessive ROS generation in cardiomyocytes results in excessive cytochrome c release which is the irreversible point in the cascade of events leading to apoptosis. The release of cytochrome c in cardiomyocytes occurs as a result of multiple events comprising ROS production, cardiolipin peroxidation, and ${Ca}^{2+}$ overload in mitochondria [45]. Recently, it has been suggested that compounds such as melatonin can prevent cardiolipin peroxidation and protect mitochondria from excessive exposure to ${Ca}^{2+}$ overload and limit reperfusion injury in the heart [47]. Another recent study has indicated the underlying mechanism of mesalazine resulting in cardiotoxic effect seems to relate to cytochrome c release, which means that mesalazine induces ROS formation leading to mitochondrial swell, mitochondrial membrane permeabilize, cytochrome c release, and eventual cardiomyocyte apoptosis [48]. However, as this study was conducted in isolated mitochondria, further studies in cells, animals, and clinical trials are still required. Overexpressed proapoptosis proteins, like Bax, cytochrome c, APAF-1, caspase 9, caspase 3, and more, play vital roles in this mitochondrion-dependent apoptosis pathway induced by oxidative stress. Specifically, excessive Bax in cytoplasm could enter mitochondria and result in disrupting mitochondrial membrane permeability, which eventually makes excessive cytochrome c release from mitochondria into cytoplasm [49]. On the other hand, a study conducted in isoproterenol-induced myocardial-infarcted rats recently revealed that α-bisabolol can abrogate the intrinsic pathway of apoptosis via reducing the expressions of these myocardial proapoptotic factors mentioned above and raising expression of antiapoptotic protein Bcl-2 [50]. Similarly, the cardiac protective effect of coccomyxagloeobotrydiformis has also been demonstrated in Mets rats with cardiovascular complications to base on facilitating the expression of Bcl-2 while suppressing the expression of Bax and cysteine protease caspase-3, protecting cardiomyocytes against apoptosis under oxidative stress [51]. Although the cardiac protective effects of α-bisabolol and coccomyxagloeobotrydiformis have been demonstrated in rats, there should be further studies to translate this therapeutic effect in humans as well as demonstrate the efficacy and safety profile in clinical treatment.

## Conclusion

Abundant experimental evidence indicates that myocardial oxidative stress generated under a variety of cardiac conditions plays a vital role in the etiopathogenesis of cardiac dysfunction in MetS. The mechanisms summarized in this review include excessive ROS-mediated mitochondrial abnormality, cardiomyocyte contractile/diastolic dysfunction, and mitochondria dependent apoptosis. Although the last few decades have seen substantial research efforts devoted to developing better therapeutic drugs to treat heart failure, the effects of currently available therapies on heart failure outcomes including mortality remain unsatisfactory. Hence, identifying the optimum intracellular nodes which aims to slow, inhibit, or even reverse the progress of cardiac dysfunction and degeneration has been one of the main challenges for optimizing therapies. Understanding the fundamental mitochondria mechanisms underlying cardiac dysfunction in MetS could not only lead to great advances in both preventative and therapeutic treatment of cardiovascular diseases but also promote the advancement of antioxidant therapies.

## Data Availability

All authors confirmed that all data and materials as well as software application support their published claims and comply with field standards.
